# Pretreatment with Apoaequorin Protects Hippocampal CA1 Neurons from Oxygen-Glucose Deprivation

**DOI:** 10.1371/journal.pone.0079002

**Published:** 2013-11-11

**Authors:** Julia A. Detert, Erin L. Adams, Jacob D. Lescher, Jeri-Anne Lyons, James R. Moyer

**Affiliations:** 1 Department of Psychology, University of Wisconsin-Milwaukee, Milwaukee, Wisconsin, United States of America; 2 Department of Biological Sciences, University of Wisconsin-Milwaukee, Milwaukee, Wisconsin, United States of America; 3 Department of Biomedical Sciences, University of Wisconsin-Milwaukee, Milwaukee, Wisconsin, United States of America; 4 Quincy Bioscience, Madison, Wisconsin, United States of America; Univ. Kentucky, United States of America

## Abstract

Ischemic stroke affects ∼795,000 people each year in the U.S., which results in an estimated annual cost of $73.7 billion. Calcium is pivotal in a variety of neuronal signaling cascades, however, during ischemia, excess calcium influx can trigger excitotoxic cell death. Calcium binding proteins help neurons regulate/buffer intracellular calcium levels during ischemia. Aequorin is a calcium binding protein isolated from the jellyfish *Aequorea victoria*, and has been used for years as a calcium indicator, but little is known about its neuroprotective properties. The present study used an *in vitro* rat brain slice preparation to test the hypothesis that an intra-hippocampal infusion of apoaequorin (the calcium binding component of aequorin) protects neurons from ischemic cell death. Bilaterally cannulated rats received an apoaequorin infusion in one hemisphere and vehicle control in the other. Hippocampal slices were then prepared and subjected to 5 minutes of oxygen-glucose deprivation (OGD), and cell death was assayed by trypan blue exclusion. Apoaequorin dose-dependently protected neurons from OGD – doses of 1% and 4% (but not 0.4%) significantly decreased the number of trypan blue-labeled neurons. This effect was also time dependent, lasting up to 48 hours. This time dependent effect was paralleled by changes in cytokine and chemokine expression, indicating that apoaequorin may protect neurons via a neuroimmunomodulatory mechanism. These data support the hypothesis that pretreatment with apoaequorin protects neurons against ischemic cell death, and may be an effective neurotherapeutic.

## Introduction

In 2009, stroke accounted for about one of every 19 deaths in the United States, making it the third leading cause of death behind only heart disease and cancer ([for review see 1]). As a result, finding ways to ameliorate injury following stroke is imperative ([for example see 2]). Much attention has been placed on the role of calcium in ischemia and possible neuroprotection by blocking its toxic effects *post*-ischemia [3].

Calcium (Ca^2+^) plays a pivotal role in various neuronal processes, including neurotransmitter release [4] and synaptic plasticity ([for review see 5]). Neurons are continuously subjected to fluctuations in intracellular Ca^2+^ as a result of ongoing activity, however excess or sustained increases in intracellular Ca^2+^ can be toxic to neurons [6]–[8]. Thus, neuronal intracellular Ca^2+^ is very tightly regulated [9], and several mechanisms exist which enable neurons to limit or control cytosolic Ca^2+^ levels [10], [11]. In particular, calcium binding proteins (CaBPs; such as calbindin, parvalbumin, and calretinin) are important for binding and buffering cytosolic Ca^2+^ [12], [13].

During ischemia, neurons are subjected to excess Ca^2+^ influx, which triggers cascades of events leading to cell death, known as excitotoxicity [7]. Since neuronal CaBPs are depleted in neurodegenerative disorders, and since neurons that express CaBPs are better able to survive an excitotoxic challenge, we reasoned that supplementing CaBPs prior to an ischemic insult will be neuroprotective.

Studies in the hippocampus have shown that the presence of CaBPs confers some protection against excitotoxic insults that normally result in cell death [14]–[17]. Interestingly, decreased levels of CaBPs are observed with advancing age [18]–[23], and in neurodegenerative disorders [14], [24]–[26], including Alzheimer’s disease [27]–[29], and Parkinson’s disease [25]. Treatments aimed at minimizing Ca^2+^ toxicity during ischemia by administering CaBPs before an ischemic insult have also had positive results [30], [31]. For example, Yenari et al. [30] treated animals with calbindin prior to inducing ischemia and found that over-expression of calbindin was neuroprotective. In addition, Fan et al. [31] treated rats with calbindin prior to ischemia and demonstrated a smaller infarct volume, better behavioral recovery, and decreased apoptosis in the calbindin-treated animals. Indeed, much research has focused on understanding the deleterious effects of stroke ([for review see 32]). Interestingly, a major risk factor for stroke is aging [1], and one prominent hypothesis of brain aging is the Ca^2+^ hypothesis of aging [33]–[35]. This hypothesis argues that an aging-related change in the ability to regulate calcium and calcium-dependent processes is a critical contributor to an increase in susceptibility to cognitive decline and neurodegenerative disorders. Given these aging-related changes in Ca^2+^, and the critical role of Ca^2+^ in ischemic cell death, much research has focused on Ca^2+^ dysregulation in both neurons [20], [36], [37] and glia [38]–[40].

Excessive intracellular Ca^2+^ accumulation following ischemia is known to potentiate cell death through excitotoxicity [41], [42]. Following an ischemic insult, Ca^2+^ accumulates within the cell through voltage-gated Ca^2+^ channels (VGCCs), through NMDA receptors, and through release from intracellular organelles [9]. Numerous studies have shown that blocking Ca^2+^ entry through NMDA receptors, VGCCs, or both in combination can be neuroprotective against ischemia [43]–[47]. Interestingly, when NMDA receptor blockers were brought to clinical trials, they failed to provide neuroprotection and they produced undesirable side effects, such as hallucinations and coma ([for review see 48]). While it is uncertain why NMDA receptor blockers failed in clinical trials, it is clear that there is a need for continued research focused on ameliorating the devastating effects of ischemic stroke. The current study targeted the use of a CaBP (AQ) and demonstrated its ability to protect neurons, possibly via its effects on various cytokines, which may provide valuable information for the development of novel neurotherapeutics.

Aequorin is a CaBP isolated from the coelenterate *Aequorea victoria.* Aequorin belongs to the EF-hand family of CaBPs, with EF-hand loops that are closely related to CaBPs in mammals [49]. In addition, aequorin has been used for years as an indicator of Ca^2+^ and has been shown to be safe and well tolerated by cells [50]. However, to date, no studies have investigated its therapeutic potential. Aequorin is made up of two components – the calcium binding component apoaequorin (AQ) and the chemiluminescent molecule coelenterazine [51]. Since the AQ portion of this protein contains the calcium binding domains, AQ was used in the present studies.

For the current experiments, we used an *in vitro* model of global ischemia in acute hippocampal brain slices. In acute hippocampal slices, OGD-induced damage is most evident in area CA1 of the hippocampus [52], similar to that seen *in vivo*. Acute hippocampal slices offer many advantages over use of cell cultures and *in vivo* models, including that the tissue morphology is relatively unchanged from the intact animal, changes in extracellular ion concentration and release of neurotransmitters are similar to that reported *in vivo*, and there is no vascular or other systemic responses that cannot be controlled *in vivo* [53]–[55]. Neuronal damage following OGD in acute slices is seen within the first 30 minutes of reperfusion [56], however, due to the short life of slices, only early changes in ischemia are able to be analyzed [57]. Because hippocampal neurons are vulnerable to cell death following ischemia [58], we tested the hypothesis that an infusion of AQ directly into the hippocampus will be neuroprotective when administered prior to an ischemic insult.

## Materials and Methods

### Subjects

Subjects were 142 adult male F344 rats (mean age 4.0±0.1 mo.; Harlan). Subjects were maintained in an Association for Assessment and Accreditation of Laboratory Animal Care (AAALAC) accredited facility on a 14 hr light–10 hr dark cycle and housed individually with free access to food and water.

### Ethics Statement

All procedures were conducted in accordance with and approved by the University of Wisconsin-Milwaukee animal care and use committee (ACUC; approved protocol 10–11 #14) and NIH guidelines, and all efforts were made to minimize suffering.

### Surgery

Rats were given ibuprofen water (15 mg/kg/day) for at least one day before and two days after surgery. On the day of surgery, rats were anesthetized with isoflurane and mounted on a stereotaxic apparatus. Under aseptic conditions, bilateral 26-gauge stainless steel guide cannulae were implanted in the dorsal hippocampus (relative to bregma: AP −3.5 mm, L ±2.6 mm, V −3.0 mm). Cannulae were secured to the skull with stainless steel screws and acrylic cement. Stainless steel caps were placed in the guide cannulae to prevent occlusion, and rats were allowed to recover at least 7 days prior to infusion.

### Intrahippocampal Infusions

The aequorin protein is made up of two components, apoaequorin and coelenterazine. The apoaequorin component (AQ) contains the EF-hands that bind Ca^2+^ [51] and thus was the component used in the current studies. Rats were given an infusion of AQ in zero Ca^2+^ artificial cerebral spinal fluid (aCSF; in mM: 124.00 NaCl, 2.80 KCl, 2.00 MgSO_4_, 1.25 NaH_2_PO_4_, 26.00 NaHCO_3_, 10.00 D-glucose, and 0.40 Na-ascorbate), which also contained 6% DMSO to facilitate neuronal uptake of AQ. Rats received bilateral infusions (0.5 µl/hemisphere) over 60 s, and the infusion cannulae remained in place for an additional 2 min to ensure diffusion away from the tip. The 33-gauge infusion cannulae were cut to extend 0.5 mm beyond the guide cannulae. To determine the dosage-dependent neuroprotection of AQ, animals were infused with 0.4, 1, or 4% AQ (w/v; Quincy Bioscience, Madison, WI [59]) in one hemisphere (counterbalanced), and the other was infused with vehicle. In addition, a subset of rats was infused with vehicle (0% AQ) in both hemispheres to serve as a control (n = 11 for each group).

### Slice Preparation

To determine the neuroprotective effect of AQ on an acute brain slice model of ischemia, 94 male F344 rats were used (mean age 4.4±0.2 mo.). Brain slices were prepared as previously described [60] from control rats (0% AQ, n = 10) or from rats infused with AQ at one of the following time points after infusion: 1 hr (n = 10), 1 day (n = 10), 2 days (n = 10), 3 days (n = 10), or 5 days (n = 5). Briefly, rats were deeply anesthetized with isoflurane, perfused through the ascending aorta with ice-cold, oxygenated (95% O_2/_5% CO_2_) sucrose-CSF (in mM: 206.00 sucrose, 2.80 KCl, 2.00 MgSO_4_, 1.25 NaH_2_PO_4_, 1.00 CaCl_2_, 1.00 MgCl_2_, 26.00 NaHCO_3_, 10.00 D-glucose, and 0.40 Na-ascorbate) and the brain rapidly removed and placed in ice-cold, oxygenated sucrose-CSF. The brain was blocked near the site of the cannula, and 400 µm thick coronal slices were cut on a temperature-controlled Vibratome as described previously [61]. Only the first 5 slices immediately posterior to the cannula placement (and devoid of any visible cannula track) were collected and used in the experiments described below. Slices were incubated on a mesh net submerged in oxygenated (95% O_2/_5% CO_2_), aCSF (composition in mM: 124.00 NaCl, 2.80 KCl, 2.00 MgSO_4_, 1.25 NaH_2_PO_4_, 2.00 CaCl_2_, 26.00 NaHCO_3_, 10.00 D-glucose, and 0.40 Na-ascorbate) at 35°C. Following a 1 hr recovery, slices were subjected to 5-min oxygen-glucose deprivation (OGD) to induce ischemia. OGD was induced by transferring the slices to a 35°C solution of fructose-CSF (in which an equimolar concentration of fructose was substituted for glucose), which was bubbled with 95% N_2_/5% CO_2_ (in which N_2_ replaced O_2_). Following the OGD, slices were transferred to a 35°C solution containing oxygenated aCSF plus 0.2% trypan blue (Sigma-Aldrich, St. Louis, MO) for 30 min reperfusion. Trypan blue penetrates dead and dying cells and stains them blue while leaving living cells unstained [62]. The slices were then briefly rinsed in room temperature, oxygenated aCSF and immediately fixed in 10% neutral buffered formalin overnight in the refrigerator. Slices were cryoprotected with 30% sucrose for a minimum of 1 day, after which they were subsectioned on a cryostat at 40 µm, mounted onto gelatin-coated slides, dehydrated in increasing steps of alcohol, and coverslipped with Permount.

### Cell Counts

The slices were examined under an upright microscope (Olympus BX51) equipped with a digital camera (Olympus DP70) and a 10X objective. Within each 40-µm subsection, a photograph was taken of the CA1 cell body layer (at the tip of the upper blade of the dentate gyrus). To avoid excessive staining due to neuronal damage as a result of the initial hippocampal slice preparation, only interior subsections were photographed for analysis. An individual blind to treatment condition then counted the number of trypan blue stained neurons located throughout the entire image. Data were counted from only one subsection. Percent neuroprotection was assessed for each animal by normalizing the data from the AQ-treated hemisphere to the vehicle-treated hemisphere.

### Western Blot Analysis

To determine how long AQ remained in the dorsal hippocampus following an infusion, 24 adult male F344 rats (mean age 4.2±0.1 mo.) were infused with 4% AQ in both hemispheres. Rats were anesthetized with an overdose of isoflurane at 1 h (n = 4), 1 d (n = 7), 2 d (n = 7), or 3 d (n = 6) after infusion, and the brain was removed, rapidly frozen on dry ice, and stored at −80°C. From each rat, two bilateral brain regions (dorsal hippocampus and ventral hippocampus; dhpc and vhpc, respectively) were dissected out and homogenized separately. Samples were centrifuged at 4000 rpm, and the supernatant removed and measured using a Bradford protein assay kit (Bio-Rad, Hercules, CA). Protein samples were normalized (50 or 150 µg/lane) and loaded for SDS-PAGE (10%). Proteins were transferred onto PVDF membranes using a semidry transfer apparatus (Bio-Rad, Hercules, CA). Membranes were then incubated for 2 hours in blocking buffer (3% nonfat dry milk) after which they were incubated in primary antibody (overnight at 4°C; 1∶5000 mouse anti-aequorin [Millipore, Billerica, MA] or 1∶1000 rabbit anti-β -actin [Cell Signaling Technology, Boston, MA]) followed by secondary antibody (90 min; 1∶5000 goat anti-mouse [Santa Cruz Biotechnology, Santa Cruz, CA] or 1∶5000 goat anti-rabbit [Millipore]). Membranes were then washed (0.05% Tween-20 in tris-buffered saline), placed in a chemiluminescence solution (Santa Cruz Biotechnology), and exposed to autoradiographic film (Hyperfilm MP). Images were taken and densitometry was performed using NIH Image J Software. A band was considered positive if the optical density value of the band (minus the background for each lane) was greater than 2 standard deviations above the mean of the ventral hippocampus bands. From this quantification, a positive band was observed in 100% of the 1 hour bands, 83% of the 1 day bands, 29% of the 2 day bands, 0% of the 3 day bands, and 0% of the vhpc lanes. Comparison was made to the ventral hippocampus because this is an adjacent brain structure that should not contain AQ, and was thus used as a negative control structure.

### Quantitative RT-PCR

Twelve male rats (each at 3.8 mo.) received unilateral infusions of 4% AQ as described above, and tissue was collected at 1 hour, 1 day, or 2 days post-infusion (n = 4 per group). Hippocampi were excised and immediately placed into TRIzol reagent (Life Technologies Corp., Carlsbad, California). Tissues were homogenized using a 25-gauge needle and syringe, and samples were stored at −80°C until RNA isolation. RNA isolation from all tissues was performed at the same time using the TRIzol method (Life Technologies Corp, Carlsbad, CA), according to manufacturer’s instructions. Isolated RNA was dissolved in 50 µl RNase free H_2_O, and RNA purity was calculated based on the absorbance ratio of 260 nm and 280 nm. An absorbance reading between 1.8 and 2.1 was considered sufficiently pure to proceed with reverse transcription. Samples presenting with a ratio less than 1.8 were further purified utilizing the Qiagen RNeasy MinElute Cleanup Kit (Qiagen, Valencia, CA) according to manufacturer’s instructions, and purified RNA was resuspended in 50 µl of RNase free H_2_O. Total RNA from all samples was reverse transcribed to cDNA using the Qiagen RT2 HT First Strand Kit-96 (Qiagen). Samples were amplified in triplicate in 96-well plates utilizing primers specific for rat IL-10 and ß-actin (RT2 qPRC Primer Assay; Qiagen) and RT^2^ SYBR Green qPCR mastermix (Qiagen) on a StepOne Real Time PCR system and software (Life Technologies Corp., Carlsbad, California). Changes in IL-10 gene expression with AQ treatment relative to vehicle treatment were calculated using the Pfaffl equation, normalizing to ß-actin expression in corresponding samples at each time-point and compared to vehicle-treated hippocampi isolated from each rat [63]. Primer efficiency was calculated based on dilution curves of two randomly selected samples for IL-10 and ß-actin. ß-Actin expression was not altered by infusion of AQ when compared to tissue infused with aCSF, indicating AQ infusion did not generally or nonspecifically affect gene transcription.

### Gene Expression Arrays

cDNA was taken from the rats used for RT-PCR (see Methods). PCR analyses focused on overall genetic markers of inflammatory cytokines and receptors, with Qiagen’s RT2 Profiler Arrays conducted as per manufacturer protocol. Briefly, 2X RT2 SYBR Green Mastermix, cDNA (see above), and RNase-free water were combined, and 25 µl of this mix was added to each well of the 96-well PCR Profiler Array well plate. Samples were run using StepOne Real Time PCR system and software, and those samples with multiple melt curves were eliminated from analysis (n = 2 excluded). One animal from the study had to be eliminated altogether, due to general variability in gene expression over two standard deviations from the mean. Gene expression changes were calculated using Qiagen’s Web-Based RT2 Profiler PCR Array Analysis Software v3.5.

### Data Analysis and Statistics

Statistical analyses were performed using Statview (v 5.0; SAS Institute, Inc., Cary, NC). An ANOVA was used to evaluate treatment effects. Fisher’s PLSD was used for *post hoc* comparisons. Data are reported as the mean ± standard error of the mean.

## Results

### Oxygen-Glucose Deprivation Results in Significant Cell Death

**Figure 1:**
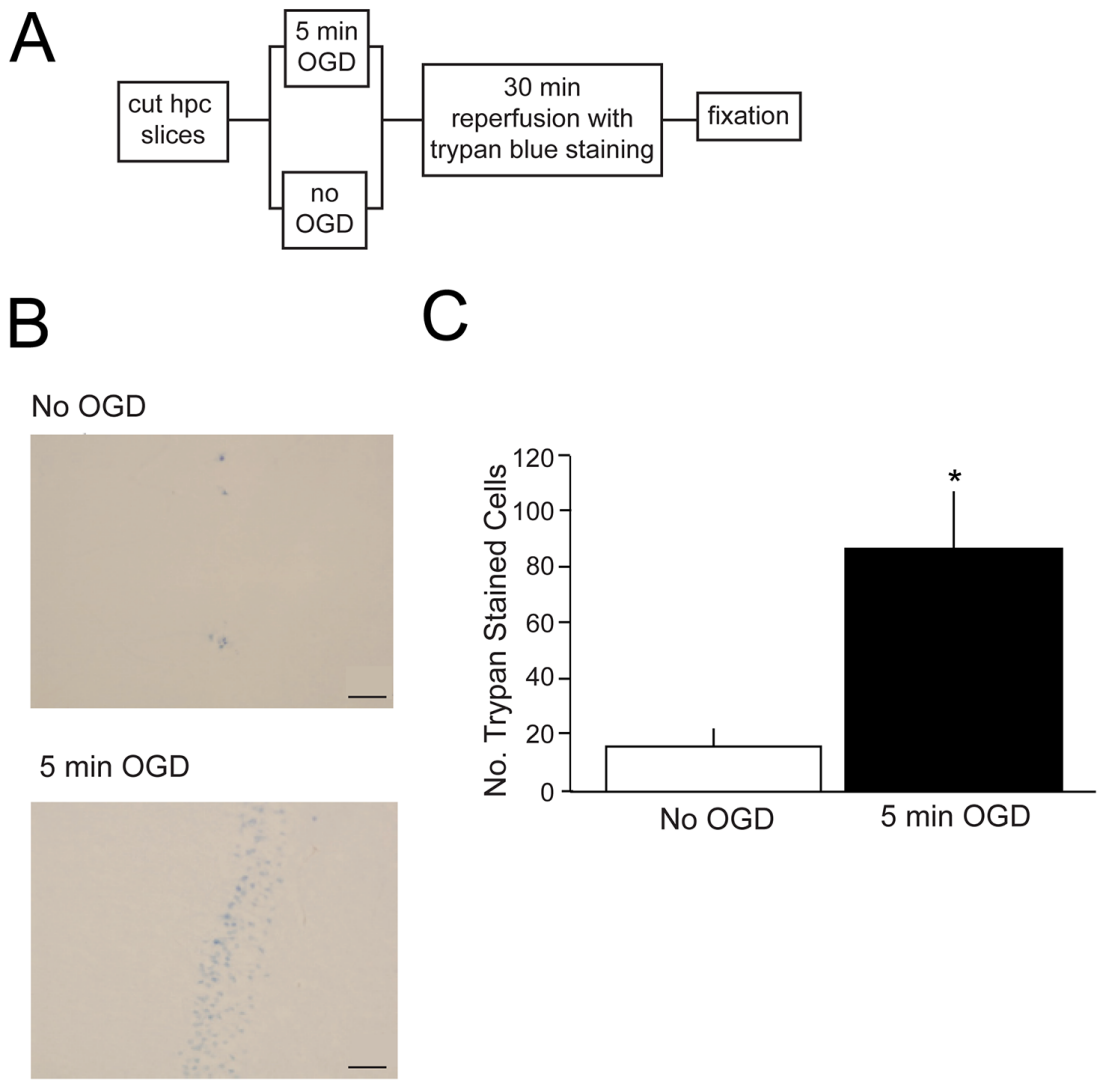
Effects of oxygen-glucose deprivation on cell death in acute hippocampal brain slices. *A*) Diagram of experimental design. Coronal hippocampal slices were incubated for 1 hr in artificial cerebral spinal fluid (aCSF). Half of the slices were transferred to the ischemic condition for 5 min of oxygen-glucose deprivation (OGD) while the other half remained normoxic (no OGD). All of the slices were then transferred to aCSF for a 30 min reperfusion and trypan blue staining. The slices were then fixed in 10% neutral-buffered formalin. *B*) Representative images of trypan blue staining in area CA1 of the hippocampus in a slice that remained normoxic (no OGD) and in a slice subjected to 5 min OGD. Notice that there is less staining in the normoxic slice compared to the OGD slice. *C*) There was a significant increase in the number of trypan blue-stained neurons in area CA1 of the hippocampus from slices that underwent 5 min OGD compared to slices that remained normoxic (*, *p*<.01).

Acute hippocampal slices were prepared, exposed to 5 min oxygen-glucose deprivation (OGD), and stained by transferring them to an oxygenated-aCSF that contained trypan blue (see methods). As can be seen in Figure 1, OGD resulted in significantly more cell death compared with control slices that did not undergo OGD. An ANOVA analyzing the average number of trypan blue-stained cells in ischemic or non-ischemic conditions demonstrated a statistically significant effect of ischemia, *F*(1, 12) = 9.65, *p*<.01. These findings are consistent with prior studies indicating that OGD results in significant cell death in CA1 region of the hippocampus [52].

### Decreased Cell Death with Apoaequorin Treatment

**Figure 2:**
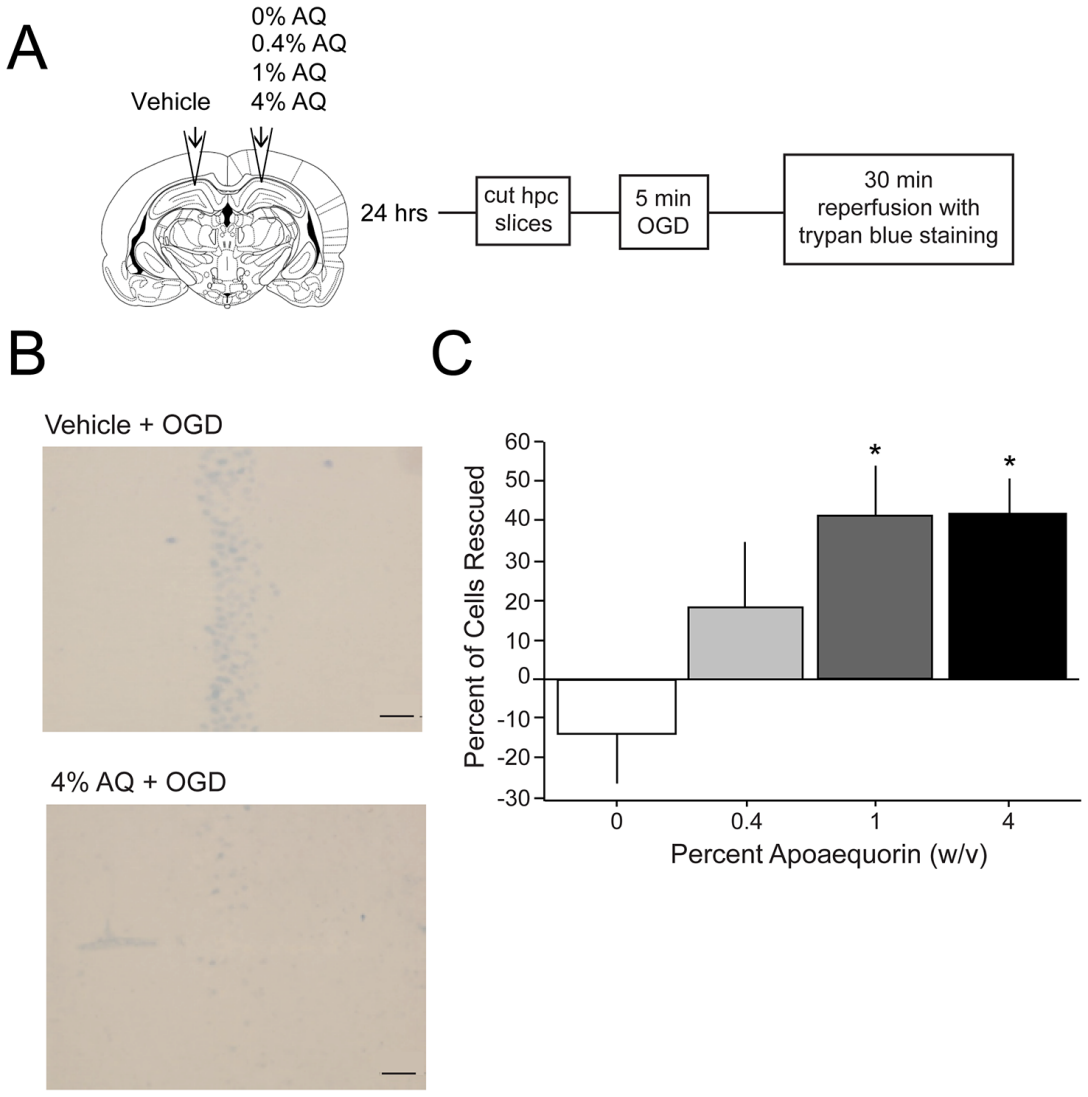
Dose-dependent effects of apoaequorin on ischemic cell death. *A*) Diagram of experimental design. Rats that were cannulated bilaterally in the dorsal hippocampus were given an infusion of 0, 0.4, 1, or 4% apoaequorin (AQ) in one hemisphere and vehicle (0% AQ) in the other hemisphere. One day following the infusion, coronal hippocampal slices were cut and incubated in artificial cerebral spinal fluid (aCSF) for 1 hr. All slices were transferred to the ischemic condition for 5 min of oxygen-glucose deprivation (OGD). Slices were then transferred to aCSF for a 30 min reperfusion and trypan blue staining. The slices were then fixed in 10% neutral-buffered formalin. *B*) Representative images of trypan blue staining in area CA1 of the hippocampus following ischemia in a vehicle-treated slice or a 4% AQ-treated slice. Notice that there is less staining in the AQ-treated slice compared to the vehicle-treated slice. C) Graph shows neuroprotection (percent of cells rescued) as a function of the dose of apoaequorin. There was significant neuroprotection in the rats treated with 1 or 4% AQ (but not 0.4% AQ) compared to the 0% AQ (vehicle; *, *p*<.01).

To examine the potential neuroprotective effects of an intra-hippocampal infusion of apoaequorin (AQ) prior to OGD, rats were infused with 0, 0.4, 1, or 4% AQ 24 hr prior to OGD (see Figure 2A). AQ was neuroprotective in a dose-dependent manner such that intra-hippocampal infusions with either 1% or 4% AQ prior to ischemia resulted in a significant increase in neuroprotection compared to vehicle (0% AQ) infusion, *F*(3, 40) = 3.61, *p*<.05 (Figure 2B&C). *Post hoc* analysis revealed that infusions of 1 or 4% AQ significantly enhanced neuroprotection relative to the 0% AQ group, *p*<.01, and that infusion of 0.4% AQ was not statistically different from any of the other groups. It was also worth noting that that amount of neuroprotection was not different between the 1% and 4% AQ treatment groups.

**Figure 3:**
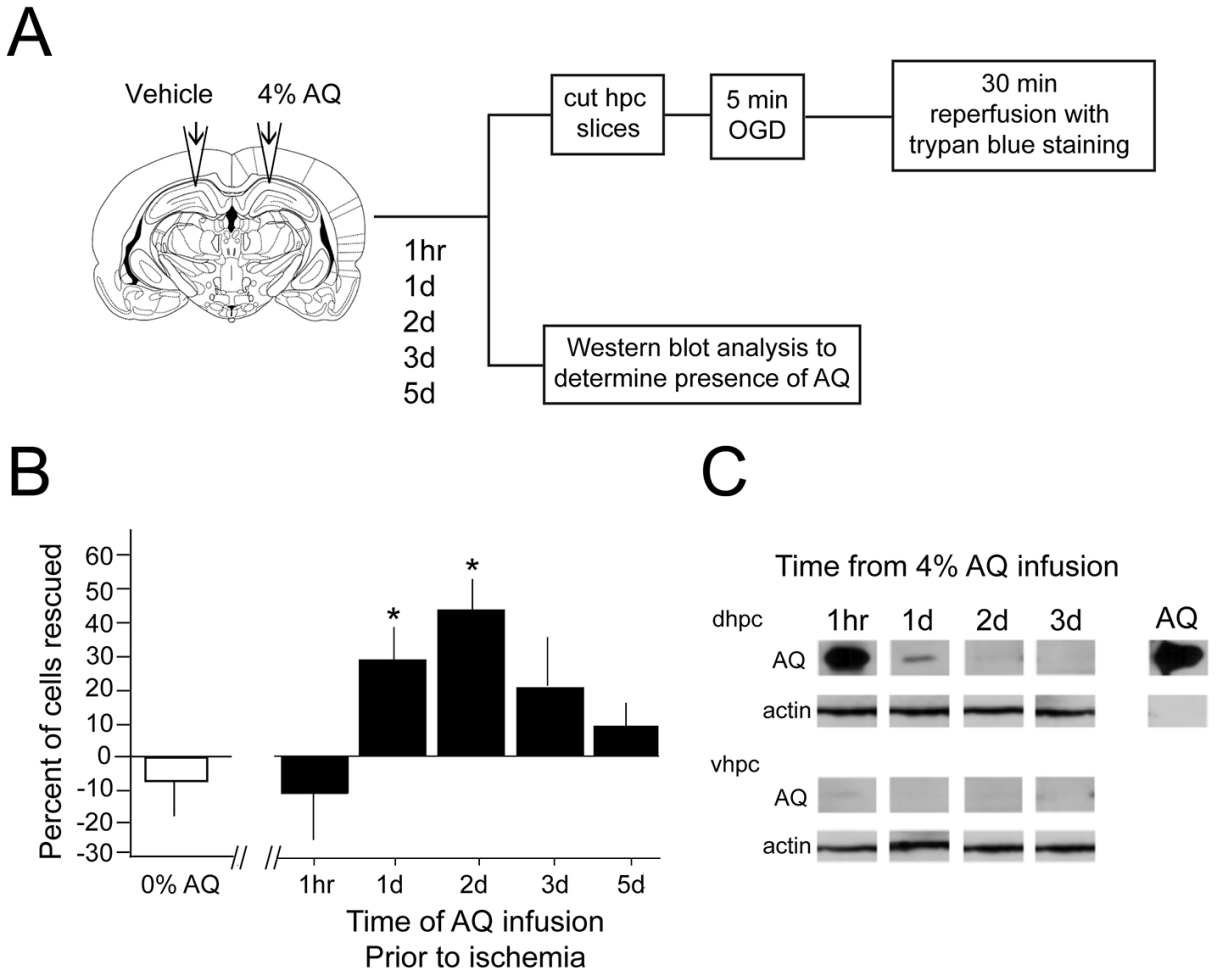
Time-dependent effects of apoaequorin on ischemic cell death. *A*) Diagram of experimental design. Rats that were cannulated bilaterally in the dorsal hippocampus were given an infusion 4% apoaequorin (AQ) in one hemisphere and vehicle (0% AQ) in the other hemisphere. Coronal hippocampal slices were cut 1 hr, 1 day, 2 days, 3 days, or 5 days post-infusion, and slices were incubated for 1 hr in artificial cerebral spinal fluid (aCSF). All slices were transferred to the ischemic condition for a 5 min oxygen-glucose deprivation (OGD). Slices were then transferred to aCSF for a 30 min reperfusion and trypan blue staining. The slices were then fixed in 10% neutral-buffered formalin. A second set of rats was given bilateral infusion of 4% AQ and the brains were removed at 1 hr, 1 day, 2 days, or 3 days post-infusion to be used for Western blotting. *B*) An infusion of 4% AQ 1 or 2 days prior to ischemia resulted in significant neuroprotection, but the neuroprotective effect was no longer evident at 3 or 5 days post-infusion. Notice that AQ is also not neuroprotective when infused just 1 hr prior to ischemia (*p* = .78). *C*). Western blot analysis of the AQ protein at 22 kD. AQ is present in the dorsal hippocampus (AQ-dhpc) at 1 hr and 1 day, but is no longer present at 3 days post-infusion. At 2 days post-infusion, a band is present in only 29% of the rats. Notice that there is never a band in the ventral hippocampus (AQ-vhpc), regardless of the infusion time. Analysis of ß-actin (45 kD) revealed no effect of protein loading at any time point in either dorsal (actin-dhpc) or ventral (actin-vhpc) hippocampus. *, *p*<.01.

To evaluate the time course over which AQ is neuroprotective, rats were infused with 4% AQ at various times (1 h, 1 d, 2 d, 3 d, or 5 d) prior to OGD (Figure 3A). One-way ANOVA indicated there was a significant effect of time on the ability of an intra-hippocampal infusion of AQ to protect neurons from a subsequent OGD, *F*(5, 49) = 3.35, *p*<.05. Post-hoc tests revealed that the neuroprotective effects of AQ required at least 1 day to emerge and that they lasted at least 2 days (*p*<.05 for each time point). No statistically significant neuroprotection was observed when slices were subjected to OGD 3 or 5 days following AQ infusion (*p* = .10 and *p = *.47, respectively).

### Western Blot Analysis of Apoaequorin

To determine how long AQ remains within the dorsal hippocampus following an intra-hippocampal infusion, AQ protein levels were measured using Western blot analysis at different times (1 h, 1 d, 2 d, or 3 d) following bilateral infusion of 4% AQ into the dorsal hippocampus. Figure 3C illustrates that within dorsal hippocampus AQ is present at 1 h and 1 d, barely visible at 2 d and no longer present by 3 d post-infusion. Thus, positive bands were observed in 100% of the 1 h, 83% of the 1 d, 29% of the 2 d, and 0% of the 3d lanes. As expected, AQ was not detected in the ventral hippocampus (vhpc), which was used as a negative control structure given its distance from the injection site (see Figure 3C). To ensure that enough protein was loaded into the gels to enable visualization of extremely faint bands, Western blots were repeated in a subset of animals, but the gels were loaded with 150 µg of protein per lane (instead of the normal 50 µg per lane). In these blots, additional bands came through in the 2- and 3-day lanes such that 57% of the 2 d and 25% of the 3 d lanes had positive bands suggesting that AQ is detectable within dorsal hippocampus for up to 3 days following dorsal hippocampal infusions. Importantly, no time-dependent changes were observed when samples were stained for β-actin, suggesting that these differences reflected time-dependent changes in the presence of AQ and not a general change in protein content (see Figure 3C).

### Cytokine and Chemokine Expression Following AQ-infusion

**Figure 4:**
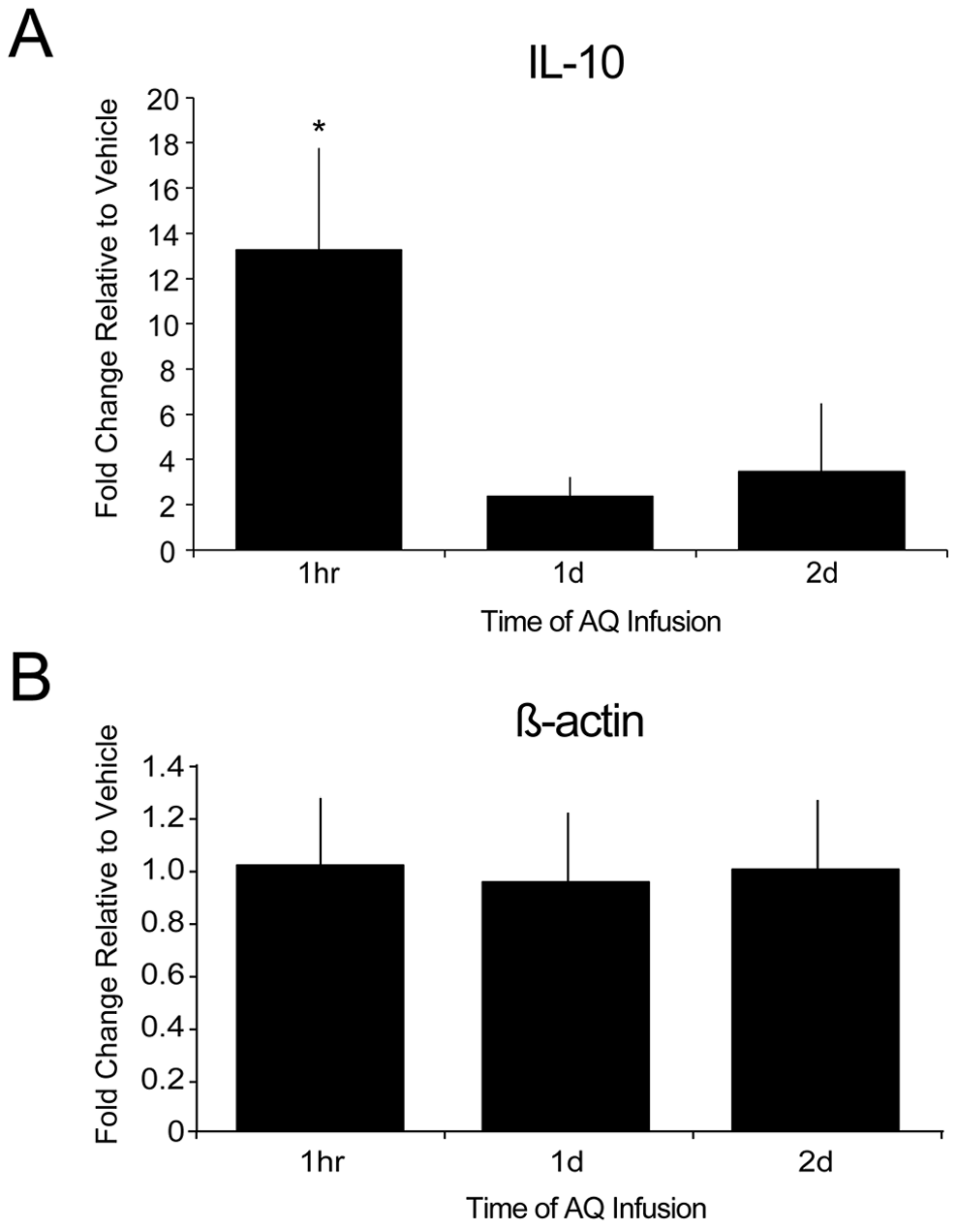
Effects of apoaequorin on interleukin-10 mRNA expression. *A*) Interleukin-10 (IL-10) mRNA expression is significantly increased 1 hour after 4% AQ was infused into the dorsal hippocampus. This statistically significant increase was transient as IL-10 mRNA expression returned to near baseline levels within 1 to 2 days, although a biologically relevant 2- to 3-fold increase was still observed. *B*) ß-actin mRNA expression did not significantly differ between 4% AQ and the vehicle-treated hemisphere (*p = *.52). For both graphs, data are expressed as fold-change from the vehicle-treated control hemisphere.

That an intra-hippocampal infusion of AQ resulted in significant neuroprotection at time points when very little protein was present suggests that AQ may trigger some cascade of events that ultimately protect neurons from an ischemic insult. One possibility is that AQ induces a pre-conditioning-like effect, resulting in reduced cell death at later time points. Ischemic pre-conditioning is a phenomenon whereby a brief ischemic episode attenuates damage caused by a subsequent more severe ischemic insult [64], [65]. Recent evidence has shown that multiple cytokines and chemokines are associated with ischemic preconditioning [6], [11], [37], [66]. Given the link between ischemic pre-conditioning and alterations in cytokine production [67], we tested the hypothesis that an infusion of AQ may lead to an increase in cytokine or chemokine expression, which may ultimately impact the ability of neurons to tolerate a later ischemic insult. RT-PCR was used to investigate mRNA changes in the anti-inflammatory cytokine interleukin-10 (IL-10), and PCR arrays were used to look at multiple gene expression changes following AQ infusion. Adult rats received an infusion of 4% AQ in one hemisphere and vehicle in the other as described (see Methods). At different times following the AQ infusion (1 h, 1 d, or 2 d later), the hippocampi were removed and quantitative RT-PCR was performed to evaluate time- and treatment-dependent changes. One-way ANOVA indicated a significant difference between the four treatment groups, *F*(3, 19) = 9.55, *p*<.0005. *Post hoc* analyses revealed that IL-10 mRNA was significantly increased 1 h after infusion in the AQ- relative to the vehicle-treated hemisphere (*p*<.001; see Figure 4A). Moreover, this AQ-induced enhancement of IL-10 expression at 1 h was significantly larger than the enhancement at 1 d (*p*<.001) or 2 d (*p*<.001). Although IL-10 expression was increased 2–3 fold at the later time points, these were not statistically significantly different from vehicle-treated hemispheres, suggesting that the significant increase in IL-10 observed at 1 hour may be due to an acute response to AQ infusion.

To investigate whether the AQ-related change in cytokine expression was restricted to IL-10 rather than being part of a more global change in mRNA expression patterns, PCR arrays were performed. Eighty-two total genes related to cytokine and chemokine responses were surveyed. Among these, 80 genes were present to varying extents in the control hemisphere and 2 genes (CCR8, chemokine receptor 8; and CRP, C-reactive protein) were not detected. Of the 80 genes that were detectable, only 16 were significantly different between AQ- and vehicle-treated hemispheres (see Table 1, data organized by response time). The majority of genes were increased at 1-hour post-AQ infusion, and thereafter decreased to or near baseline levels by 1 day. Of the 8 that were significantly upregulated at 1 hour, only one remained elevated through the 2-day post-infusion time point, Chemokine ligand 10 (CXCL10). Six genes were not significantly upregulated at 1 hour but were upregulated at 1-day post-AQ infusion. Of these six, only two did not remain elevated at 2 days – Chemokine ligand 11 (CXCL11) and Interleukin-1 receptor type II (IL-1rII). Only two genes were significantly upregulated exclusively at the 2 days post-AQ infusion time point – Chemokine receptor 1 (XCR1) and Complement component 3 (C3). These results indicate that an infusion of AQ into the dorsal hippocampus has a dramatic effect on cytokine and chemokine mRNA expression at both short- and long-term time points.

**Table 1:** Fold change in genes following 4% AQ infusion, grouped by response time.

	Time From AQ Infusion		
Fast Responders (within 1 hour)	1 Hour	1 Day	2 Days
Chemokine ligand 1 (CXCL1)	19.36^†^	1.77	−1.81
Chemokine ligand 3 (CCL3)	20.07^†^	8.85*	1.15
Chemokine ligand 4 (CCL4)	33.89^†^	6.20*	1.68
Chemokine ligand 10 (CXCL10)	9.59*	6.27	8.45*
Interleukin 1 alpha (IL-1α)	36.63^†^	1.23	1.25
Interleukin 1 beta (IL-1β)	32.46^†^	8.94*	1.43
Interleukin-10 (IL-10)	5.26*	4.27	3.14
Tumor necrosis factor (TNF-α)	23.15^†^	4.64	−1.29
**Slower Responders (within 1 day)**			
CD40 ligand	1.62	17.91^†^	7.15*
Chemokine ligand 9 (CXCL9)	1.27	26.46^†^	13.47*
Chemokine ligand 11 (CXCL11)	4.47	15.22^†^	3.84
Chemokine receptor 3 (CXCR3)	1.17	35.51^†^	11.66 ^†^
Interleukin 1 receptor, type II (IL-1rII)	2.09	6.90*	−1.16
Interleukin 2 receptor, beta (IL-2rβ)	1.74	11.92^†^	6.70*
**Slowest Responders (within 2 days)**			
Chemokine receptor 1 (XCR1)	−2.04	3.19	8.81*
Complement component 3 (C3)	2.07	3.93	10.11^†^

Numbers represent fold change from vehicle-infused hemisphere (*p<.05; ^†^ *p*<.01).

## Discussion

The current study demonstrates that the calcium binding protein apoaequorin (AQ) is neuroprotective in a time- and dose-dependent manner when administered prior to ischemic injury. Intra-hippocampal infusion of either 1% or 4% AQ resulted in significantly fewer dead or dying neurons as compared to animals infused with control (see Figure 2). This neuroprotection was time-dependent, in that it took up to 1 or 2 days to develop and it subsided by 3 to 5 days. Neuroprotection may involve a pre-conditioning like effect, whereby an AQ infusion modulates cytokine and chemokine expression, which subsequently protects neurons from oxygen-glucose deprivation (OGD).

Previous studies have suggested a neuroprotective role for CaBPs. For example, neurons that contain the CaBP calbindin are more resistant to excitotoxic and ischemia-related injuries than neurons that lack calbindin [68]. In addition, some studies have noted that calbindin expression increases following traumatic brain injury and ischemia [69]–[71], indicating that calbindin may be increased to maintain Ca^2+^ homeostasis and protect against excitotoxicity. Likewise, using either gene therapy or protein transduction, overexpression of CaBPs prior to ischemia has also been found to be neuroprotective [30], [31]. In contrast, that calbindin is present in both the dentate gyrus (an area resistant to ischemic cell death) and CA1 (an area vulnerable to cell death) has been used as an argument against a role for calbindin in neuroprotection [72]. Finally, others have reported that recovery from ischemia is enhanced in calbindin knockout mice [73]. Since these were not inducible knockouts, it is possible that other compensatory mechanisms played a role in the observed neuroprotection.

Studies examining the effect of artificial calcium chelators (e.g., BAPTA-AM, EGTA, etc…) on excitotoxicity have had mixed results, with some studies finding neuroprotection [45], [74], [75] and others finding enhanced vulnerability to cell death [76]–[78]. Nikonenko et al. [45] demonstrated neuroprotection in rat organotypic hippocampal slice cultures following OGD in slices treated with EGTA, BAPTA, Mibefradil, Kurtoxin, Nickel, Zinc, and Pimozide. On the contrary, Abdel-Hamid and Baimbridge [76] loaded cultured hippocampal neurons with the calcium chelator BAPTA-AM and found enhanced glutamate excitotoxicity in those neurons. The authors’ suggest that the presence of artificial calcium chelators interferes with normal Ca^2+^-dependent mechanisms that prevent Ca^2+^ influx into the cell. These opposing results may be due to a number of factors, including the mode of inducing excitotoxicity, the type of Ca^2+^ chelator used, or the use of cultured neurons as compared to acute brain slices.

Interestingly, when the AQ protein was most readily detected in the dorsal hippocampus, at 1-hour post-infusion, neuroprotection was not observed (see Figure 3). Although it is unknown how or whether AQ enters the cell, the current study used DMSO with AQ for infusions, which is used to transport drugs across membranes. Thus, it is likely that AQ had the opportunity to enter cells. Moreover, the centrifugation process for the Western blot samples was designed to isolate intracellular components of the cell (by centrifuging at a low speed), and AQ’s presence in these samples strongly suggests its presence within the cells. Although significant neuroprotection was evident at 1 and 2 days post-infusion, much less AQ was evident in dorsal hippocampus (Figure 3C), suggesting that neuroprotection did not merely result from immediate effect of AQ binding Ca^2+^. Rather, the data suggest that neuroprotection results from a cascade of events caused by the AQ infusion. Since the neuroprotective effects were observed at 1 and 2 days post-infusion when the protein was barely present or not detected (but not at 1 hour when AQ expression was at its highest), this cascade is likely to be due to other AQ-triggered mechanisms, including a pre-conditioning-like effect post-infusion. This type of an effect would take time to develop [79], and would explain why neuroprotection was not immediately observed (e.g., 1 hr post-infusion). Preconditioning may also explain why robust neuroprotection was observed at 1 or 2 days post-infusion, despite lower detection of the protein at these time points. While the exact mechanisms are currently unknown, studies have implicated cytokines and chemokines in preconditioning [80].

To investigate whether the observed neuroprotection following AQ infusion is due to a preconditioning-like effect, we measured changes in IL-10 mRNA, an anti-inflammatory cytokine known to be involved in preconditioning [11], [81], [82]. A statistically significant increase in IL-10 mRNA was observed 1 hour after infusion. Although not statistically significant, a biologically significant (>2-fold) increase in IL-10 mRNA continued to be observed for up to 2 days following AQ infusion (see Figure 4A). Anti-inflammatory cytokines can act by recruiting cell populations that are protective through cytokine secretion, which in turn prevent or down-regulation the induction of a damaging pro-inflammatory immune response, actively protecting against future insult [83]. The increased IL-10 expression at 1-hour post-AQ infusion may be serving as a protective primer for the upcoming OGD insult such that 1–2 days later, the brain is fully primed and better able to withstand an ischemic insult. This effect is short-lived such that by 3–5 days post-AQ infusion, little to no neuroprotection is evident.

Given that an increase in IL-10 mRNA at 1 hour post-AQ infusion suggests a preconditioning-like effect, multi-gene PCR arrays were used to evaluate the effects of AQ on the expression of a wide variety of cytokines and chemokines (see Table 1). These studies revealed that AQ infusion differentially regulates, in a time-dependent manner, expression of a number of cytokines and cytokine receptor genes compared to the vehicle-treated hemisphere. Of the 82 total genes examined in the array, 16 were significantly upregulated following infusion of AQ. Within these 16, a time-dependent effect was evident, such that 8 were rapidly upregulated immediately following AQ infusion whereas the remaining 8 were upregulated only after a 1- or 2-day delay.

Of the cytokines that were upregulated post-AQ infusion, effects of preconditioning have been examined in only four: (1) interleukin-1ß (IL-1ß), (2) IL-10, (3) tumor necrosis factor-α (TNF-α), and (4) complement component 3 (C3). All four of these cytokines have been shown to be increased following preconditioning [84]–[88]. IL-1ß, a pro-inflammatory cytokine, has been shown to increase within 6 hours after preconditioning [86] after which it returns to baseline within 3–4 days [67]. This is consistent with the present study, which demonstrates a rapid increase in IL-1ß mRNA followed by a return to baseline levels by 2 days post-AQ (Table 1). While IL-1ß is a pro-inflammatory cytokine, moderate increases can be neuroprotective [87]. Likewise, IL-10 has also been shown to rapidly increase following preconditioning, with a fairly quick return to baseline [84]. Here we show using both quantitative RT-PCR (Figure 4) and PCR arrays (Table 1) that IL-10 is significantly upregulated at 1 hr post-AQ infusion. IL-10 has been shown to decrease the release of TNF-α [89] and reduce brain injury following focal ischemia in rats [90]. Following preconditioning, TNF-α is rapidly upregulated [85], persists for up to 2 days, and is no longer detected after 3–4 days [67]. The current experiments demonstrate an increase in TNF-α gene expression at 1 hour, but not at 1 or 2 days post-AQ infusion. C3 was significantly upregulated 24 hours following lipopolysaccharide (LPS) preconditioning [88]. Here, a significant increase in C3 gene expression was observed at 2 days after AQ-infusion. Activation of the complement host defense system, including C3, has been shown to have both damaging and protective effects [91]. Taken together, these data indicate that the increase in IL-1ß, IL-10, TNF-α, and C3 in the current experiment may be one reason for the neuroprotective effects of AQ-infusion.

While only four of the upregulated cytokines have been examined in preconditioning, almost all of the 16 have been examined following cerebral ischemia. Only chemokine ligand-9 (CXCL9), chemokine ligand-11 (CXCL11), and chemokine receptor-1 (XCR1) have not been, to our knowledge, previously examined with their relations to cerebral ischemia. Of the other cytokines, all have been shown to increase following ischemia, except Interleukin-2 receptor, beta (IL-2rß). Under normal conditions, IL-2rß is found within the cell membrane of hippocampal CA1 pyramidal neurons. Following ischemia, IL-2rß not only decreases within CA1, but it also translocates from the cell membrane to the cytoplasm and nucleus [92]. How some cytokines function following ischemia likely depends upon their expression patterns, which may influence when and whether they are neuroprotective or not. For example, CD40 ligand plays a role in inflammation and tissue injury, and it is upregulated following focal ischemia [93]. However, CD40 ligand also protects neurons from neuronal stress and deficiency in CD40 ligand results in neuronal dysfunction, indicating that CD40 ligand is important for general neuronal function [94]. The present data indicate a significant increase in CD40 ligand at both 1 and 2 days post-AQ infusion. This sustained increase in CD40 ligand may contribute to the time course of our observed neuroprotection. Although beyond the scope of the present study, it will be important (and the data suggest worthwhile) to further assess the neuroprotective effects of AQ over a longer time frame using an *in vivo* model of ischemia.

In conclusion, the current experiments support the hypothesis that AQ protects neurons against ischemia when administered directly to the brain prior to an ischemic insult. These effects are both dose- and time-dependent such that a single intra-hippocampal infusion of AQ protects neurons from OGD for up to 2 days. Moreover, AQ infusions activated cytokine and chemokine gene expression in a manner similar to those seen with ischemic preconditioning. Thus, pretreatment with AQ may be an effective way to protect neurons against ischemic stroke by acting as a chemical preconditioning agent.
